# 4-week aerobic exercise training regulates systemic macrophage polarization in obese mice

**DOI:** 10.7717/peerj.20604

**Published:** 2026-01-15

**Authors:** JingAo Qin, HaiBin Zhang, XinPeng Gao, Nan Zhang, Xin Zhang, Jeong-sun Ju

**Affiliations:** 1 Physical Education, Yulin University, Yulin, Shaanxi, China; 2 Sports Science, The University of Suwon, Hwaseong-si, Gyeonggi-do, Republic of South Korea

**Keywords:** Aerobic exercise training, Macrophage polarization, Obesity, Inflammation, Pro-inflammatory, Anti-inflammatory, High-fat diet

## Abstract

**Background:**

Obesity is accompanied by chronic low-grade inflammation, largely driven by imbalances in macrophage polarization. While pro-inflammatory M1 macrophages accumulate in adipose tissue and circulation, contributing to insulin resistance and metabolic disruption, alternatively activated M2 macrophages exert anti-inflammatory and tissue-protective effects. Exercise is widely recognized as a non-pharmacological strategy to improve metabolic health; however, the extent to which short-term aerobic training influences systemic macrophage polarization in obesity is not fully understood. This study examined whether a 4-week aerobic exercise intervention alters systemic macrophage polarization in diet-induced obese mice and explored its role in attenuating obesity-related inflammation.

**Methods:**

Male C57BL/6J mice (8 weeks old) were fed either a standard chow diet (Ch) or a high-fat diet (HF; 60% kcal from fat) for 12 weeks. Following obesity induction, HF-fed mice were assigned to either a sedentary (HF-Sed) or exercise-trained (HF-Exe) group. The training protocol involved treadmill running at moderate intensity, performed twice daily, 5 days per week, for 4 weeks. Plasma concentrations of M1-associated markers (TNF-α, IFN-γ , IL-1β, IL-6) and M2-associated markers (IL-10, Arg1, CD163) were measured by an enzyme-linked immunosorbent assay (ELISA). Statistical differences were analyzed using analysis of variance (ANOVA) with *post hoc* testing.

**Results:**

After 12 weeks of high-fat feeding, mice exhibited approximately 20% higher body weight than chow controls, confirming obesity induction. Four weeks of exercise training did not significantly reduce body weight but improved metabolic indices, including plasma glucose and insulin sensitivity. HF-Sed mice displayed elevated circulating M1 cytokines, whereas HF-Exe mice had significantly lower levels of IL-6, and TNF-α. Conversely, exercise enhanced M2-associated markers, including IL-10, Arg1, and CD163. Thus, aerobic training shifted systemic macrophage polarization away from a pro-inflammatory toward an anti-inflammatory profile, independent of substantial weight loss.

**Conclusion:**

Short-term aerobic exercise is sufficient to promote M2 macrophage polarization and dampen systemic inflammation in obese mice. These findings underline the rapid immunomodulatory potential of exercise and support its role as an effective non-pharmacological approach to counteract obesity-related inflammation and metabolic dysfunction.

## Introduction

Obesity has become a major global public health issue affecting the quality of life and health of hundreds of millions of people (Arena et al., 2021). Obesity significantly elevates the risk for type 2 diabetes, cardiovascular diseases, many cancers, and kidney complications. The pathophysiological consequences of obesity extend beyond metabolic dysregulation: a persistent, low-grade inflammatory state underpins many obesity-related comorbidities, including insulin resistance, endothelial dysfunction, and organ dysfunction and pathology (Chen et al., 2025). Obesity is also strongly associated with chronic inflammation (Diane et al., 2016). Research indicates that obesity can lead to a chronic inflammatory state of adipose tissue *in vivo*, mainly due to excessive release of proinflammatory factors caused by macrophage polarization to M1 type (Tulp, 2024). This inflammatory response not only affects metabolic function, but also promotes insulin resistance, which exacerbates the development of obesity-related diseases (He et al., 2017).

Recently, macrophage polarization has attracted considerable attention as a central research topic in the study of obesity. Macrophage polarization refers to the process where macrophages adopt functionally distinct phenotypes in response to their microenvironment. The classical M1 phenotype is induced by signals such as interferon-gamma (IFN-γ) and lipopolysaccharide (LPS). It exhibits a pro-inflammatory profile, characterized by the secretion of cytokines including tumor necrosis factor (TNF-α), interleukin-6 (IL-6), and interleukin-1 beta (IL-1β), as well as the generation of reactive species. In contrast, the alternative M2 phenotype, stimulated by interleukin-4 (IL-4) and interleukin-13 (IL-13), release anti-inflammatory factors such as interleukin-10 (IL-10) and promote tissue repair and anti-inflammatory responses (Li et al., 2023; Yao, Xu & Jin, 2019). In obesity, there is a pronounced shift toward M1-like macrophage polarization in adipose tissue-evidenced by increased infiltration of TNF-α, IL-6, and monocyte chemoattractant protein-1 (MCP-1)-secreting macrophages-contributing to chronic inflammation and insulin resistance. In contrast, M2 macrophages dominate in lean adipose tissue and support metabolic homeostasis. Obesity-induced imbalance in macrophage polarization can lead to a persistent inflammatory state that exacerbates metabolic disorders (Casuso & Huertas, 2021). Recent advances have illuminated the key role of immunometabolism—the inherent metabolic state of macrophages—in regulating not only their polarization but also their capacity for cytokine production, migration, and tissue remodeling. New evidence points to metabolic reprogramming (including altered glucose, fatty acid, ketone, and amino acid oxidation) as a determinant of macrophage fate and function in adipose tissue and beyond, and as a potential therapeutic target for obesity-linked inflammation (Makassy, Williams & Karwi, 2025).

Aerobic exercise is widely recognized as an effective means of improving obesity and related diseases (Nieman, 2020). Recent studies reveal that regular exercise not only improves adiposity and metabolic outcomes, but also exerts profound immunomodulatory effects, influencing the polarization of both tissue-resident and circulating macrophages (Dousdampanis, Aggeletopoulou & Mouzaki, 2025). Exercise is increasingly recognized as a key regulator of immune function, in part through its effects on macrophage polarization (Voskoboynik, McCulloch & Sahoo, 2024). Acute bouts of exercise can transiently activate pro-inflammatory pathways and increase M1-like macrophage activity, which facilitates tissue remodeling and adaptation to physiological stress. However, long-term or regular exercise training promotes a sustained shift toward the M2 phenotype, characterized by anti-inflammatory signaling, improved insulin sensitivity, and enhanced tissue repair (Callegari, Rocha & Lliveira, 2023). This shift is mediated by exercise-induced myokines, including IL-6 and IL-10, as well as improved oxidative and metabolic conditions within skeletal muscle and adipose tissue. Importantly, this polarization balance counteracts the chronic low-grade inflammation associated with obesity and metabolic disorders, highlighting the therapeutic potential of exercise in immune-metabolic regulation (Goh, Goh & Abbasi, 2016; Zhang et al., 2024).

While much mechanistic work has focused on local tissues (*e.g.*, skeletal muscle, adipose tissue, cardiovascular system), emerging evidence suggests exercise may also influence circulating monocytes and macrophage-lineage cells, indicating potential systemic immunomodulatory effects. Flow-cytometric profiling shows that exercise and physical activity can shift monocyte subset distributions (classical CD14^++^CD16^−^, intermediate CD14^++^CD16^+^, non-classical CD14^+^CD16^++^) and alter polarization-related surface markers (*e.g.*, CD80 for M1-like, CD163/CD206 for M2-like) (Blanks et al., 2020). In humans, moderate training has been reported to suppress M1-associated signals and enhance M2-associated signals in circulating monocytes, potentially *via* PPARγ pathways, aligning with improved insulin sensitivity (Ruffino et al., 2016). Beyond cellular phenotyping, soluble macrophage activation markers measured in plasma (particularly sCD163 and sCD206 shed from macrophage membranes) have emerged as tractable systemic readouts across diseases. These markers reflect distinct activation and shedding mechanisms and are increasingly studied as noninvasive indices of macrophage activity (Nielsen et al., 2019; Nielsen et al., 2020).

Exercise-related macrophage polarization has primarily been examined at the local level in tissues such as skeletal muscle and adipose tissue, whereas studies investigating the systemic effects of exercise on macrophage polarization remain limited. This study aims to investigate the effects of four weeks of aerobic exercise on systemic macrophage activation, assessed in blood *via* soluble markers (sCD163 and sCD206) and cytokines, to provide insights into the immune-modulating benefits of exercise and inform strategies for the prevention and management of obesity-related inflammation and metabolic disorders.

## Materials & Methods

### Animals and treatment

Twenty-four eight-week-old male wild-type C57BL/6J mice (weighing 17–19 g) were purchased from SPF Biotechnology Co., Ltd (Beijing, China) and used in this study. Animals were housed four per a polycarbonate cage (27.5 cm length × 23 cm width × 12 cm height) in a temperature (∼22 °C), humidity 50%–60% and a 12:12-h light-dark cycle with free access to food and water. All protocols for animals use and euthanasia were approved by Animal Experimental Ethics Committee of Yulin University (approval number: YULLPZ-2025-001). All experimental procedures were conducted in accordance with institutional guidelines for animal care. Humane endpoint criteria were established for euthanizing animals prior to the planned end of the experiment; however, all animals remained healthy, and no early euthanasia was required. At the conclusion of the experiment, all surviving animals were humanely euthanized following institutional guidelines. The standard chow diet and the high-fat diet were obtained from Xiaoshu Youtai Company (Beijing, China). The normal chow diet was based on the AIN-93M international formula, providing 15% of energy from protein, 9% from fat, and 76% from carbohydrates. The high-fat diet was formulated according to the D12492 international standard and provided 20% of energy from protein, 60% from fat, and 20% from carbohydrates.

**Figure 1:**
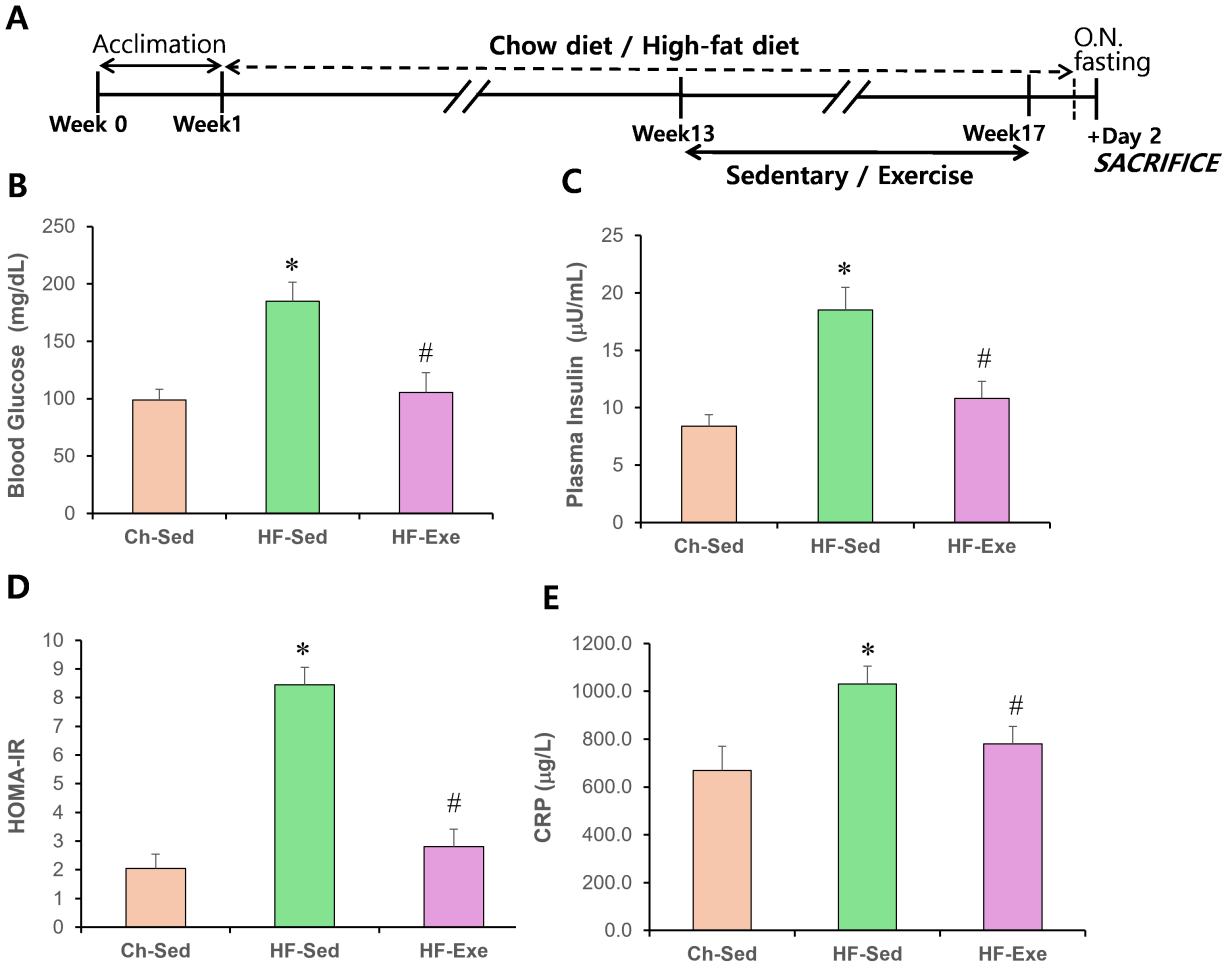
Effects of a 4-week exercise intervention on plasma glucose, insulin, and HOMA-IR in high-fat diet–fed mice. (A) Experimental schematic. Male C57BL/6J mice were maintained on either normal chow or a high-fat diet (60% kcal from fat) ad libitum for 12 weeks. After 12 weeks, high-fat diet–fed mice were divided into sedentary or treadmill exercise groups. Following 4 weeks of intervention, mice were fasted overnight, and blood was collected 48 h after the final exercise session. (B) Blood glucose (mg/dL) and plasma insulin (μU/mL) levels were measured. (C) HOMA-IR was calculated as: (fasting insulin × fasting glucose)/405. Data are presented as mean ± SD; *n* = 8 per group. * *p* < 0.05 *vs.* Ch-Sed; # *p* < 0.05 *vs.* HF-Sed. HOMA-IR, Homeostatic Model Assessment for Insulin Resistance; Ch-Sed, chow-sedentary; HF-Sed, high-fat sedentary. Effects of a 4-week exercise intervention on serum CRP level in high-fat diet–fed mice. (E) Serum CRP level. Elevated CRP are indicative of systemic metabolic inflammation. Data are presented as mean ± SD; *n* = 8 per group. * *p* < 0.05 *vs.* Ch-Sed; # *p* < 0.05 *vs.* HF-Sed. O.N. fasting, overnight fasting; CRP, C-reactive protein; Ch-Sed, chow sedentary; HF-Sed, high-fat sedentary; HF-Exe, high-fat exercise.

After a 1-week acclimation period, the animals were initially assigned randomly to two groups: chow diet fed (*n* = 8) and high-fat diet-fed (*n* = 16). After 12 weeks of feeding, the body weights of high-fat diet-fed mice were 20% higher than the average weight of the chow diet-fed group. Over 20% increase in body weight over chow-fed controls is a typical threshold for defining obesity in high-fat diet-induced mouse models (Ren et al., 2022). The high-fat diet–fed mice were then randomly divided into two groups: sedentary and exercise. Accordingly, three groups were established: chow-fed sedentary control (Ch-Sed, *n* = 8), high-fat diet–fed sedentary (HF-Sed, *n* = 8), and high-fat diet–fed exercise intervention (HF-Exe, *n* = 8). Mice in the HF-Exe group performed treadmill running exercise for four weeks (Fig. 1A). The sample size of eight mice per group was chosen based on previous studies investigating skeletal muscle atrophy and interventions in mice, which showed that 6–10 animals per group are sufficient to detect statistically significant differences in muscle mass, histological features, and functional outcomes. Using eight mice per group provides adequate statistical power while minimizing the number of animals used, in accordance with the principles of the Replacement, Reduction, and Refinement (3Rs) (Rogers et al., 2025; Wang et al., 2023b).

### Body weight and food intake

Body weight was measured once a week, and the average value for each group was calculated. Food was provided twice a week, and its weight was recorded each time before feeding. Before the next feeding, the remaining food was weighed to determine food intake. Food intake was measured twice a week, and both the amount consumed per animal, and the daily food consumption were calculated.

### Training program

After successful establishment of the obese mouse model, the Ch-Sed group was maintained on a normal diet, while the HF-Sed and HF-Exe groups continued to receive a high-fat diet. Mice in the HF-exe group performed treadmill exercise twice daily at 9:00 a.m. and 4:00 p.m. The running exercise training was conducted 5 days per week using a modified treadmill protocol as described previously (Wang et al., 2023a). The training protocol was as follows: during the first week, mice ran on a treadmill (0° incline), starting with a 5-minute warm-up at 6 m/min, followed by 20 min of running at 10 m/min, and ending with a 5-minute cool-down at six m/min. From the second week onward, the warm-up speed was increased to nine m/min, the main training consisted of 20 min at 12 m/min, and the cool-down was performed at nine m/min for 5 min. The exercise intervention lasted for four weeks.

### Animal sacrifice and blood sample collection

To avoid the influence of acute exercise, blood samples were collected 48 h after the final running session. Mice were fasted for 12 h and anesthetized by intraperitoneal injection of 4% sodium pentobarbital. After confirming anesthesia, approximately 0.8 mL of blood was collected from the orbital sinus, and the mice were subsequently sacrificed by cervical dislocation. The blood samples were allowed to clot at room temperature for 30 min and then centrifuged at 3,000 × g for 5 min at 4 °C. The serum supernatant was then collected and stored at −80 °C for subsequent analyses.

### Measurement of plasma parameters

After a 12-hour fasting period, blood was collected from the tail vein of each animal, and the concentrations of plasma triglycerides (mg/dl), total cholesterol (mg/dl), and lactate (mmol/l) were measured using an Accutrend Plus analyzer (Roche Diagnostics, Rotkreuz, Switzerland). Plasma glucose (mg/dl) and β-hydroxybutyrate (Mm) were measured using a FreeStyle Optium Neo kit (Abbott, Illinois, USA). Plasma insulin was assayed by enzyme-linked immunosorbent assay (ELISA) (Abcam, Cambridge, UK, ab277390).

### Enzyme-linked immunosorbent assay

The surface marker of M2 macrophages was quantified using an ELISA. The ELISA was performed as previously described in Oh et al. (2017), with minor modifications. Briefly, 96-well microplates (Corning 3690) were coated with 30 µL of coating buffer (0.1 M sodium carbonate–sodium bicarbonate, pH 9.5; 1.59 g Na_2_CO_3_ and 7.13 g NaHCO_3_ dissolved in one L of deionized water) containing a 1:500 dilution of the capture antibody (anti-CD163, Abcam, ab272407). Plates were incubated overnight at 4 °C, after which the coating solution was removed, and wells were blocked with 50 µL of blocking buffer (1% bovine serum albumin (BSA) in phosphate-buffered saline (PBS)) for 1 h at room temperature (RT). Serum samples (diluted 1:101) were premixed with an equal volume of dilution buffer (PBS, pH 7.4, containing 0.05% Tween-20 and 1% BSA), and 30 µL of the mixture was added to each well. After incubation for 1 h at RT, wells were washed three times with PBS with Tween-20 (PBST). Subsequently, 30 µL of the detection antibody (anti-CD163, Abcam, ab272407) diluted in the same buffer was added to each well and incubated for 1 h at RT. Following three additional washes with PBST, 30 µL of tetramethylbenzidine (TMB) substrate solution (1-Step™ Ultra TMB-ELISA, Thermo Scientific, 34028) was added to each well and incubated for 30 min at RT. The reaction was stopped by adding 30 µL of 1 N H_2_SO_4_, and absorbance was measured at 450 nm using a microplate reader (BIOBASE EL10A automatic microplate reader).

The levels of other cytokines were measured using mouse ELISA kits (NEIMIAN, JIANGSU, CHINA) and the specific product numbers are as follows: the specific product numbers are as follows: C-reactive protein (CRP) (MM-0074M2), IL-1β (MM-0040M2), IL-6 (MM-0163M2), IL-10 (MM-0176M2), TNF-α (MM-0132M2), IFN-γ (MM-0182M2), and enzyme Arg1 (Abcam, Cambridge, UK, ab269541). The ELISA assays were performed according to the manufacturer’s instructions.

### Statistical analysis

The data were analyzed using SPSS version 26.0 (SPSS Inc., Chicago, IL, USA). Results are expressed as mean ± standard deviation (SD). Differences among groups were assessed using one-way analysis of variance (ANOVA). A significance level of 0.05 was set, and when a statistically significant difference was detected, Tukey’s *post hoc* test was performed.

## Results

### Effects of 4-week exercise intervention on physical characteristics and blood parameters in HFD-fed mice

At the end of the 16-week treatment, significant differences in average body weight were observed among the groups. The HF-Sed group exhibited an approximately 21% increase compared with the Ch-Sed group, whereas the HF-Exe group did not differ significantly from the Ch-Sed group (Table 1). These results indicate that both the high-fat diet–induced obesity model and the exercise training protocol were successfully established in this study. The average daily food intake per mouse (g/day/mouse) did not differ significantly among the three groups (*p* > 0.05). However, the food efficiency ratio (FER) differed significantly between groups. The HF-Sed group exhibited a higher FER compared with the Ch-Sed group, whereas the FER of the HF-Exe group did not differ significantly from that of the Ch-Sed group (Table 1, *p* < 0.05). Moreover, exercise reduced the FER in the HF-Exe group compared with the HF-Sed group. These results indicate that the high-fat diet promoted greater weight gain per unit of food intake, while exercise mitigated this effect.

Following the 16-week treatment, both liver weight and total visceral fat weight were significantly higher in the HF-Sed group compared to the Ch-Sed group (Table 1, *p* < 0.05). The 4-week exercise intervention effectively attenuated these increases, significantly reducing liver weight and total visceral fat weight. The HF-Sed group showed significant elevations in blood triglyceride and total cholesterol levels compared with the Ch-Sed group (*p* < 0.05). Importantly, the 4-week exercise intervention effectively attenuated these increases, as evidenced by the reduced blood lipid levels in the HF-Exe group (*p* < 0.05). In contrast, no significant differences were observed in blood β-hydroxybutyrate (β-HB) or lactate levels among the three groups (Table 1, *p* > 0.05).

### Effects of 4-week exercise training on HOMA-IR and CRP in high-fat diet–fed mice

The effects of a high-fat diet, with or without a 4-week exercise training, on blood glucose and insulin levels in fasted mice were examined. As expected, obese mice fed the high-fat diet were hyperglycemic (Fig. 1B) and had elevated blood insulin levels (Fig. 1C). In contrast, high-fat diet–fed obese mice that underwent exercise training had normal blood glucose and insulin levels (Fig. 1C). The Homeostasis Model Assessment of Insulin Resistance (HOMA-IR) score in the HF-Sed group was 8.45 ± 0.6, indicating insulin resistance (HOMA-IR > 3.0), compared with 2.01 ± 0.5 in the Ch-Sed group and 2.81 ± 0.6 in the HF-Exe group (Fig. 1D). These results indicate that exercise training corrects high-fat diet–induced hyperglycemia and hyperinsulinemia.

**Table 1:** Body weight, food intake, and plasma parameters (*n* = 8 per group). Values are means ± SD; *n* = 8 per group. FER, Food efficiency ratio = body weight gain (g)/total food intake × 100. β-HB, beta-hydroxybutyrate.

	Ch-Sed	HF-Sed	HF-Exe
Final Body weight (g)	29.2 ± 2.61	35.3 ± 1.43^*^	32.5 ± 1.02^#^
Food intake (g/day/mice)	3.7 ± 0.5	3.3 ± 0.7	4.1 ± 0.6
FER (BWG/FI*100)	5.54 ± 0.64	9.87 ± 0.76^*^	6.04 ± 0.55^#^
Liver weight (g)	1.19 ± 0.22	1.95 ± 0.32^*^	1.27 ± 0.21^#^
Total visceral fat (g)	1.1 ± 0.6	2.7 ± 0.5^*^	0.95 ± 0.7^#^
β-HB (mmol/L)	1.57 ± 0.3	1.65 ± 0.2	1.48 ± 0.4
Lactate (mmol/L)	1.38 ± 0.1	1.51 ± 0.3	1.43 ± 0.2
Triglyceride (mg/dL)	95.5 ± 6.8	152.0 ± 7.8^*^	98.9 ± 15.4^#^
Cholesterol (mg/dL)	103.1 ± 5.5	168.4 ± 9.7^*^	119.3 ± 8.5^#^

**Notes.**

* *p* < 0.05 *vs.* Ch-Sed

# *p* < 0.05 *vs.* HF-Sed.

Ch-Sed, chow sedentary; HF-Sed, high-fat sedentary; HF-Exe, high-fat exercise.

CRP is commonly used as a blood inflammatory marker and is associated with chronic low-grade inflammation, such as that observed in obesity and metabolic syndrome. A 16-week high-fat diet (60% fat) significantly increased CRP level in the blood of the HF-Sed group compared with the Ch-Sed group (Fig. 1E, *p* < 0.05). Notably, a 4-week exercise intervention significantly attenuated this elevation (Fig. 1E, *p* < 0.05). These results indicate that aerobic exercise training acts as a potent anti-inflammatory stimulus.

### 4-week exercise training reduces M1 macrophage markers in blood of high-fat diet–fed mice

**Figure 2:**
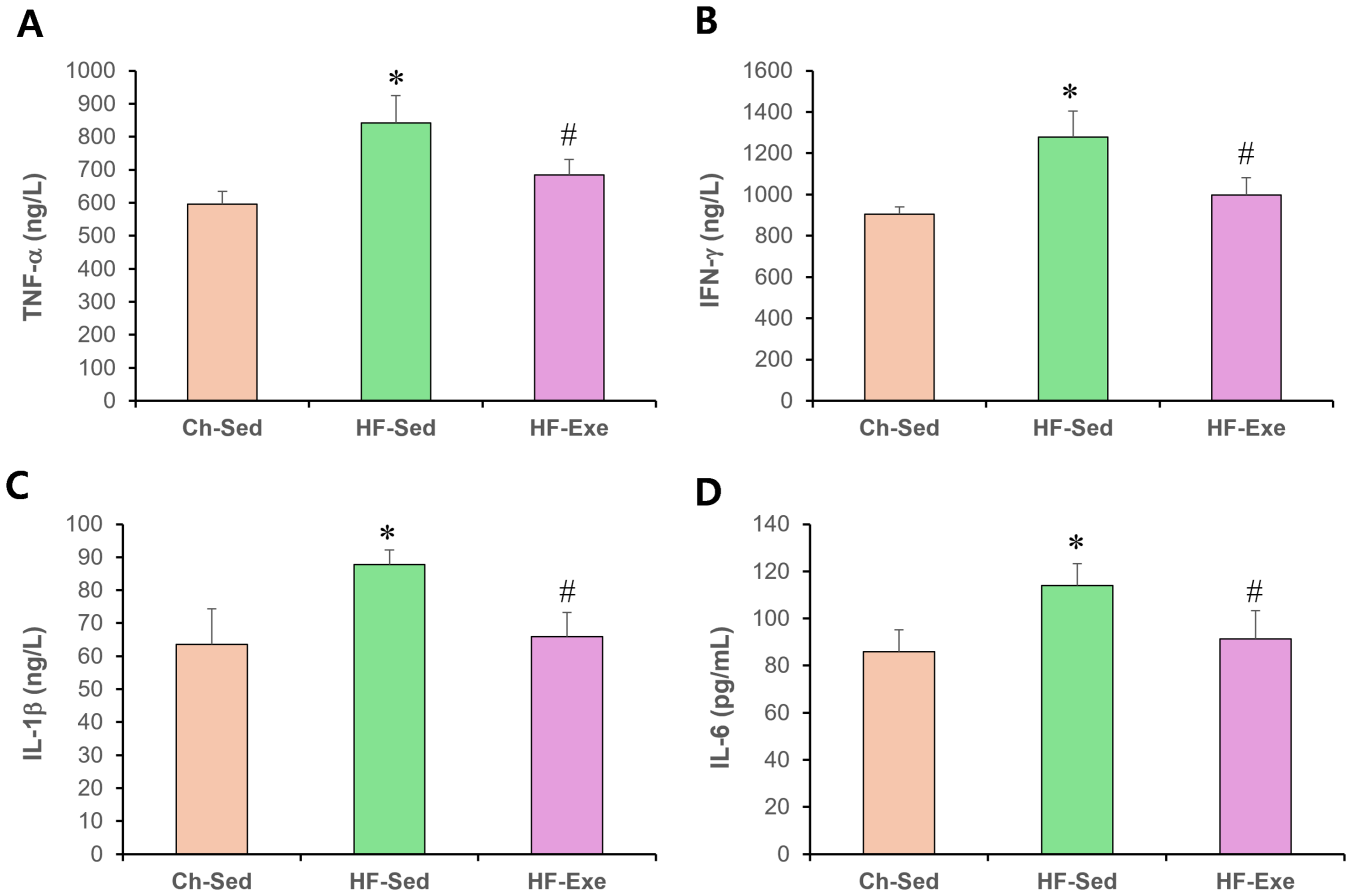
Effects of a 4-week exercise intervention on M1 macrophage–related proinflammatory factors in high-fat diet–induced obese mice. Male C57BL/6J mice were maintained on either normal chow or a high-fat diet (60% kcal from fat) ad libitum for 12 weeks. After 12 weeks, high-fat diet–fed mice were divided into sedentary or treadmill exercise groups. Following 4 weeks of intervention, mice were fasted overnight, and blood was collected 48 h after the final exercise session. The blood samples were allowed to clot at room temperature for 30 min and then centrifuged at 3,000 × g for 5 min at 4 °C. The serum supernatant was then collected. Serum concentrations of (A) TNF-α, (B) IFN-γ, (C) IL-1β, and (D) IL-6 were quantified using enzyme-linked immunosorbent assays (ELISA). ELISA assays were performed according to the manufacturer’s instructions using mouse cytokine ELISA kits. A 4-week exercise regimen shifted macrophage polarization by suppressing proinflammatory signaling driven by M1 macrophages in obese mice. Data are expressed as mean ± SD; *n* = 8 per group. * p < 0.05 *vs.* Ch-Sed; # *p* < 0.05 *vs.* HF-Sed. TNF-a, Tumor necrosis- a; IFN- g, Interferon-g; IL, Interleukin; Ch-Sed, chow sedentary; HF-Sed, high-fat sedentary; HF-Exe, high-fat exercise.

To evaluate systemic pro-inflammatory M1 macrophage polarization, we measured plasma levels of TNF-α, IFN-γ , IL-1β, and IL-6 by ELISA in Ch-Sed, HF-Sed, and HF-Exe groups (Fig. 2). Sixteen weeks of high-fat diet feeding significantly increased all four M1 macrophage–related inflammatory markers in the HF-Sed group compared with the Ch-Sed group, by approximately 41%, 41%, 38%, and 32%, respectively (Figs. 2A–2D, *p* < 0.05). Importantly, a 4-week exercise intervention effectively reduced these elevated markers by approximately 19%, 22%, 25%, and 20%, respectively (Figs. 2A–2D, *p* < 0.05). Collectively, these results demonstrate that exercise training attenuates high-fat diet–induced systemic pro-inflammatory M1 macrophage polarization.

### 4-week exercise training increases M2 macrophage markers in blood of high-fat diet–fed mice

**Figure 3:**
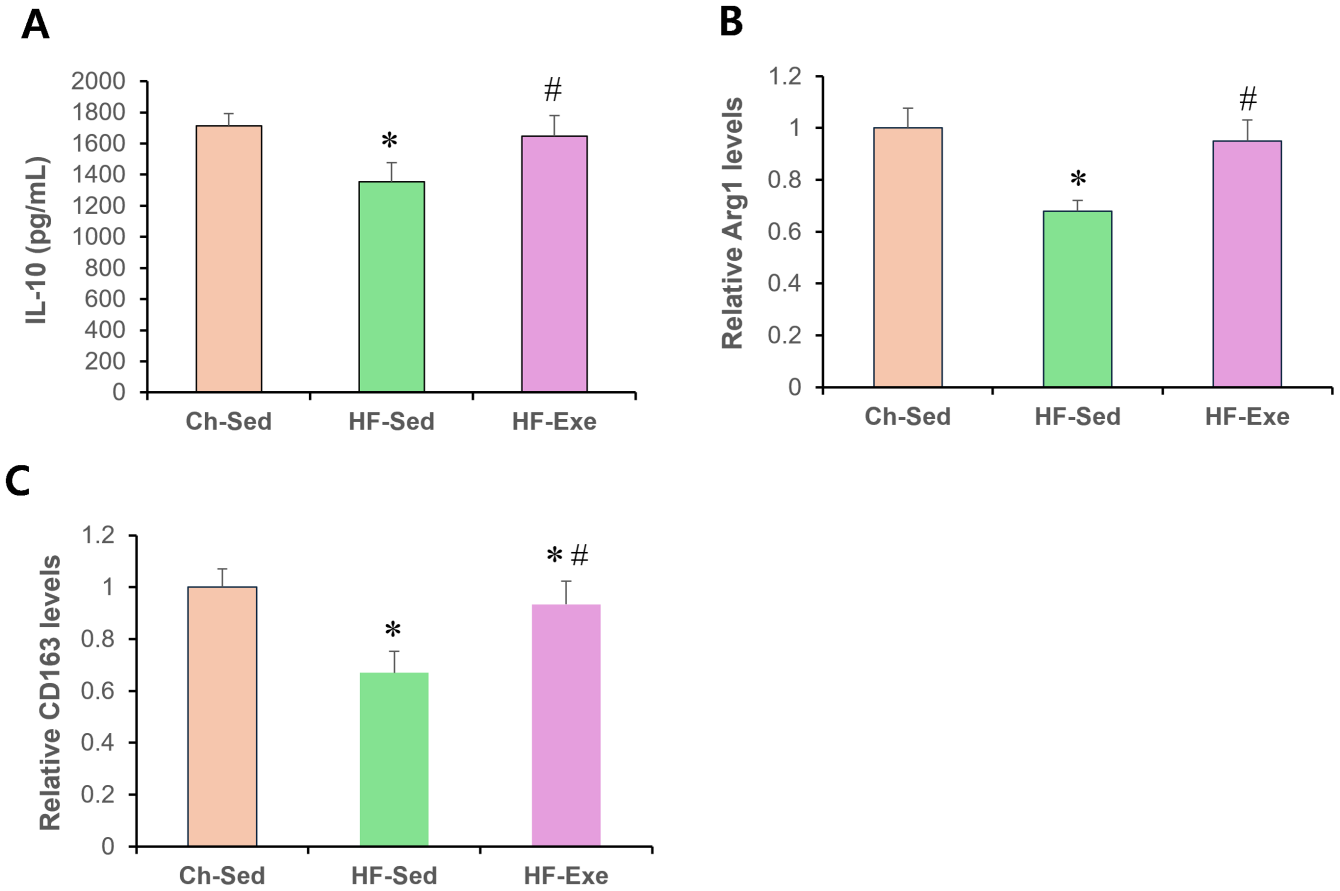
Effects of a 4-week exercise intervention on M2 macrophage–related anti-inflammatory factors in high-fat diet–induced obese mice. Male C57BL/6J mice were maintained on either normal chow or a high-fat diet (60% kcal from fat) ad libitum for 12 weeks. After 12 weeks, high-fat diet–fed mice were divided into sedentary or treadmill exercise groups. Following 4 weeks of intervention, mice were fasted overnight, and blood was collected 48 h after the final exercise session. The blood samples were allowed to clot at room temperature for 30 min and then centrifuged at 3,000 × g for 5 min at 4 °C. The serum supernatant was then collected. Serum concentrations of (A) IL-10, (B) Arg1, and (C) CD163 were measured by enzyme-linked immunosorbent assays (ELISA). All cytokines were quantified using commercial mouse ELISA kits as described in the Methods section. A 4-week exercise program influenced macrophage polarization by promoting anti-inflammatory signaling associated with M2 macrophages in obese mice. Data are presented as mean ± SD; *n* = 8 per group. * *p* < 0.05 *vs.* Ch-Sed; # *p* < 0.05 *vs.* HF-Sed. IL, interleukin; Arg1, Arginase 1; CD163, Cluster of Differentiation 163; Ch-Sed, chow sedentary; HF-Sed, high-fat sedentary; HF-Exe, high-fat exercise.

Next, we assessed systemic anti-inflammatory M2 macrophage polarization by measuring serum IL-10, Arg1, and CD163 levels in Ch-Sed, HF-Sed, and HF-Exe groups (Fig. 3). Sixteen weeks of high-fat diet feeding significantly reduced all three M2 macrophage–related inflammatory markers in the HF-Sed group compared with the Ch-Sed group, by approximately 30%, 21%, 32%, and 33%, respectively (Figs. 3A–3D, *p* < 0.05). Notably, a 4-week exercise intervention effectively restored these reductions, increasing the markers by approximately 74%, 27%, 40%, and 39%, respectively (Figs. 3A–3D, *p* < 0.05). Together, these results demonstrate that exercise training promotes systemic anti-inflammatory M2 macrophage polarization in high-fat diet-fed obese mice.

## Discussion

In the present study, we investigated the effects of a 4-week aerobic exercise intervention on systemic inflammatory markers and macrophage polarization in diet-induced obese mice. The major findings were that (1) obesity led to a clear pro-inflammatory state, evidenced by elevated CRP, TNF-α, IFN-γ , IL-1β, and IL-6, alongside reduced M2-associated markers (IL-10, Arg1, CD163); and (2) short-term aerobic exercise training effectively mitigated the obesity-associated inflammatory profile, lowering systemic M1 markers (TNF-, IL-6, IFN-, IL-1β) while enhancing anti-inflammatory M2-associated markers (IL-10, Arg1, CD163). These results demonstrate that short-term aerobic training can systemically attenuate the chronic low-grade inflammation associated with obesity by rebalancing macrophage-associated cytokine levels. While body weight remained unchanged, the exercise-induced reduction in visceral fat may have contributed to these systemic anti-inflammatory effects, suggesting that the benefits are not solely dependent on whole-body weight loss. Consistent with previous studies, we found that obesity promoted a shift toward M1 macrophage dominance, characterized by increased circulating pro-inflammatory cytokines including TNF-α, IL-6, and IL-1β. M1 macrophages contribute to insulin resistance by impairing insulin signaling pathways and sustaining tissue inflammation. In contrast, the decline in M2-related markers such as IL-10 and CD163 reflects a loss of anti-inflammatory regulation and tissue repair mechanisms. These findings support the concept that disruption of the M1/M2 balance plays a critical role in obesity-associated metabolic dysfunction (He et al., 2017; Casuso & Huertas, 2021).

Our data show that aerobic exercise partially attenuates obesity-induced skewing of the systemic inflammatory profile. Specifically, exercise reduced M1-associated pro-inflammatory markers (TNF-α, IL-6, IFN-γ , IL-1β) while increasing M2-associated markers (IL-10, Arg1, CD163). The observed systemic effects do not permit conclusions regarding the reversal of macrophage polarization within specific tissues, which remains to be investigated. The decreases in visceral fat weight after exercise may partly explain the anti-inflammatory effects, offering a more refined view of the intervention’s weight-independent mechanisms. These shifts mirror adaptations reported in previous studies showing that regular exercise attenuates inflammatory signaling and improves metabolic flexibility (Kawanishi et al., 2013; Ziegler et al., 2019). A key mechanism may involve exercise-induced production of myokines, such as IL-6, which (when released in the context of exercise rather than adiposity) exerts anti-inflammatory actions by stimulating IL-10 and inhibiting TNF-α expression. Additionally, improvements in oxidative metabolism and insulin sensitivity likely provide a permissive environment for M2 polarization, supported by enhanced fatty acid oxidation and reduced glycolytic dependence.

While earlier work primarily focused on tissue macrophages in adipose tissue or skeletal muscle, our findings extend this knowledge by documenting systemic changes in circulating macrophage markers. In particular, the restoration of soluble M2 markers (*e.g.*, CD163) suggests that aerobic training influences not only local tissue inflammation but also circulating immune cell phenotypes, providing a potential biomarker for exercise-induced immune adaptation. This systemic perspective supports the growing evidence that exercise modifies the monocyte/macrophage compartment in blood, which may contribute to whole-body improvements in metabolic health.

The ability of aerobic exercise to induce systemic M2 polarization, independent of major weight loss, has important therapeutic implications. This suggests that improvements in inflammation and metabolic function can be achieved before substantial changes in body mass, reinforcing exercise as a frontline intervention in obesity management. Moreover, our findings highlight macrophage polarization as a mechanistic target that may mediate many of the anti-inflammatory benefits of exercise.

Several limitations of the present study should be acknowledged. First, although our conclusions regarding macrophage polarization are based on circulating cytokine profiles, these data do not directly demonstrate changes in macrophage phenotypes at the cellular level. Complementary analyses, such as flow cytometry of blood monocytes or histological assessment of tissue-resident macrophages, would be required to confirm polarization status more conclusively. Second, the study included only male mice; therefore, sex-specific responses should be examined in future work. Third, we evaluated a single exercise modality and duration; it remains to be determined whether different intensities, durations, or resistance training produce similar systemic effects.

Future studies should combine systemic profiling with tissue-specific analysis and mechanistic assays to determine how exercise regulates macrophage metabolism (glycolysis *vs.* oxidative phosphorylation) and signaling pathways (*e.g.*, PPARγ , AMPK, or HIF-1α). Translational studies in humans will also be needed to confirm whether soluble M2 markers such as CD163 can serve as reliable biomarkers of exercise-induced immune adaptation in obesity.

## Conclusions

In summary, our findings suggest that a short-term aerobic exercise intervention can shift macrophage polarization toward an anti-inflammatory M2 phenotype, reducing systemic inflammation in obese mice. These results highlight the potential of exercise as an immunomodulatory strategy to mitigate obesity-related inflammation and improve metabolic health, although further studies are needed to confirm applicability in humans.

##  Supplemental Information

10.7717/peerj.20604/supp-1 Supplemental Information 1 Raw data Raw data of Arg1, blood parameters, body weight, CRP, Food consumption, Liver weight, IFN-gamma, CD163, IL-1, IL-2, IL-6, IL-10, TNF-alpha

10.7717/peerj.20604/supp-2 Supplemental Information 2 Author checklist

## Additional Information and Declarations

### Data Availability

The following information was supplied regarding data availability: The raw data are available in the Supplemental File.
